# 3D printing in fracture treatment: Current practice and best practice consensus

**DOI:** 10.1007/s00113-022-01159-y

**Published:** 2022-07-11

**Authors:** Christian Fang, Leyi Cai, Gabriel Chu, Rahat Jarayabhand, Ji Wan Kim, Gavin O’Neill

**Affiliations:** 1 Queen Mary Hospital, The University of Hong Kong, Hong Kong SAR, China; 2 First Affiliated Hospital, Wenzhou Medical University, Wenzhou, China; 3 United Christian Hospital, Hong Kong SAR, China; 4 Bhumibol Adulyadej Hospital, Bangkok, Thailand; 5 Asan Medical Centre, University of Ulsan College of Medicine, Seoul, Korea (Republic of); 6 National University Hospital, Singapore, Singapore

**Keywords:** Intra-articular fractures, Fracture fixation, Anatomic models, Patient-specific instruments, Organization and administration, Intraartikuläre Frakturen, Frakturfixation, Anatomische Modelle, Patientenspezifische Instrumente, Organisation und Administration

## Abstract

The use of 3D printing in orthopedic trauma is supported by clinical evidence. Existing computed tomography (CT) data are exploited for better stereotactic identification of morphological features of the fracture and enhanced surgical planning. Due to complex logistic, technical and resource constraints, deployment of 3D printing is not straightforward from the hospital management perspective. As a result not all trauma surgeons are able to confidently integrate 3D printing into the daily practice. We carried out an expert panel survey on six trauma units which utilized 3D printing routinely. The most frequent indications are acetabular and articular fractures and malalignments. Infrastructure and manpower structure varied between units. The installation of industrial grade machines and dedicated software as well as the use of trained personnel can enhance the capacity and reliability of fracture treatment. Setting up interdisciplinary jointly used 3d printing departments with sound financial and management structures may improve sustainability. The sometimes substantial logistic and technical barriers which impede the rapid delivery of 3D printed models are discussed.

**Supplementary Information:**

The online version of this article (10.1007/s00113-022-01159-y) also contains details of the data collected during the survey.

## Abstract (de)

Der Einsatz des 3D-Drucks zur Versorgung von Frakturen wird durch klinische Evidenz gestützt. Vorhandene CT-Daten werden für eine verbesserte stereotaktile Identifizierung der morphologischen Frakturmerkmale und eine verbesserte Operationsplanung genutzt. Aufgrund komplexer logistischer, technischer Schwierigkeiten und Ressourcenbeschränkungen ist die Nutzung des 3D-Drucks aus Sicht des Krankenhausmanagements nicht einfach. Infolgedessen können nicht alle Unfallchirurgen den 3D-Druck in ihre tägliche Praxis integrieren. In 6 unfallchirurgischen Kliniken, die diesen in der Routine nutzen, wurde eine Expertenbefragung durchgeführt. Die häufigsten Indikationen sind Acetabulum- oder andere Gelenkfrakturen und Fehlstellungen. Infra- und Personalstruktur variierten zwischen den Einheiten. Die Installation von Industriemaschinen und dedizierter Software sowie der Einsatz von geschultem Personal können die Kapazität und Zuverlässigkeit der Frakturversorgung erhöhen. Die Errichtung von interdisziplinär gemeinsam genutzten 3D-Druck-Abteilungen mit einer soliden Finanz- und Managementstruktur kann die Nachhaltigkeit verbessern. Die z. T. erheblichen logistischen und technischen Barrieren, die die schnelle Lieferung von 3D-gedruckten Modellen behindern, werden diskutiert.

A fast, cost-effective and high-quality three-dimensional (3D) printing strategy in (acute) fracture management is extremely important. The aim of this paper is to systematically present the experiences gained from the application of 3D printing processes and to formulate pragmatic recommendations for their use.

## Introduction

The goal of using 3D printing in the management of fractures is to improve surgical quality and efficiency [1]. The use of 3D printed anatomical models for tactile preoperative and intraoperative assessment enhances the recognition of pathoanatomical details and intuitive execution of the surgical plan. Improved outcomes regarding surgical duration [1, 2], blood loss [1, 2], fluoroscopy use [1] and fracture reduction [1, 2] are reported. Typical indications include complex articular fractures [3], acetabulum fractures [2], and fractures in areas of unusual or unfamiliar anatomy [4, 5]. Patient-specific osteotomy [6] and reduction jigs are useful in the management of various posttraumatic deformities [7].

## Methodology

The practice of 3D printing for orthopaedic trauma in six Asiatic institutions were surveyed using a structured format through the authors’ personal connections. Two phone interviews were carried out with each co-author using a Delphi method regarding distinctive aspects in their current practice and later a consensus was drawn after a second phone review.

The topic headings chosen in this article are defined by the production workflow and the administrative structure. For the production pathway the headings were indications, infrastructure, image acquisition, digital workflow and production. For administration, the headings were organization and funding, future developments and research. For each of these aspects, the current methods of the centres were summarized in a best practice consensus.

The main points of the interviews are summarized in the appendix table (online). Five of the six centres have a total hospital case load of more than 100 cases and two have an excess of 200 cases per year when counting all specialities. Concerning only fracture-related cases, this is between 12 and 50% of the total cases. Therefore, at the hospital level, fracture-related 3D printing is one of their major services.

## Indications

### Current practice.

**Fig. 1:**
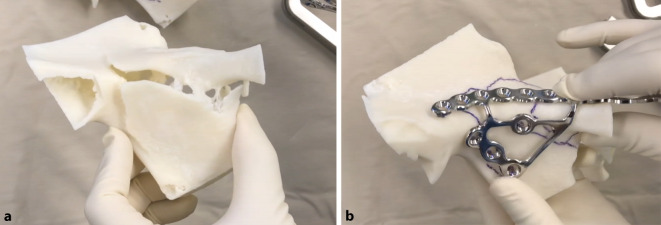
**a** Model of an acetabular fracture and **b** model of the mirrored contralateral side for implant planning and precontouring

**Fig. 2:**
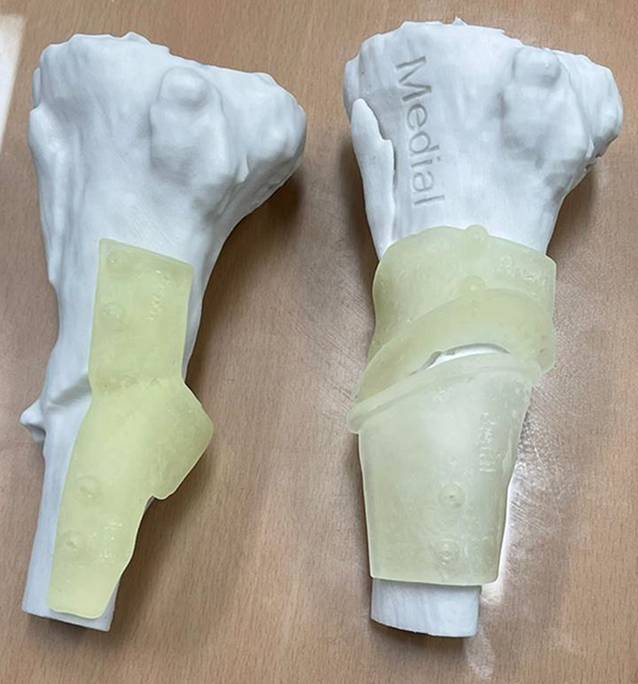
Correction of a malalignment in a proximal tibia fracture in two steps using a 3D printed osteotomy template (*right*) and reduction guide (*left*)

There are small variations in indications depending on patients’ demographics and surgeon preference. The top indications for 3D printing are visualization of complex articular (Fig. 1) and acetabulum fractures. Shaft fractures are less commonly indicated. Patient-specific osteotomy guides (Fig. 2) and shoulder replacement guides are common in three centres. Patient-specific fracture fixation implants are used in two centres.

### Consensus.

Three-dimensional models are useful for acetabulum fractures [8], complex periarticular fractures [9, 10] and malunions [11]. They are used in preoperative planning, implant contouring, surgical simulation [13], patient communication [4], and training [14].

Implants can be preshaped and contoured for each patient using 3D printed models

**Fig. 3:**
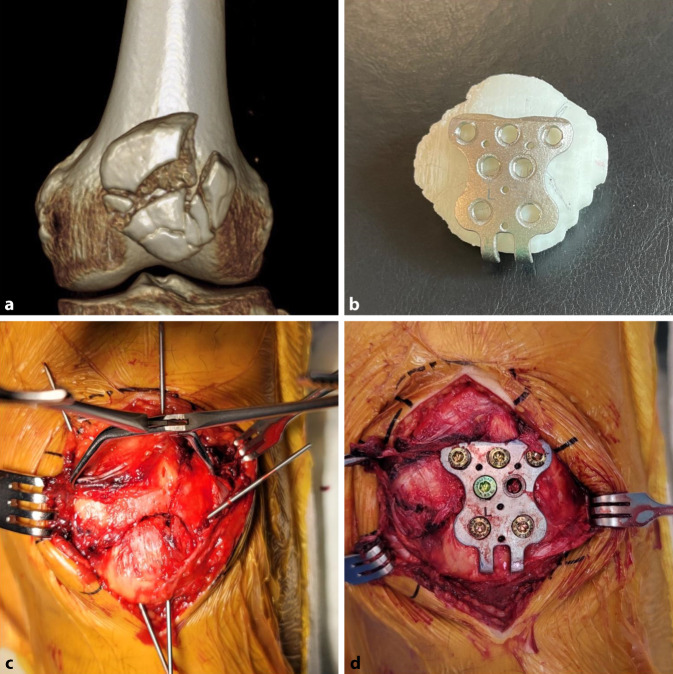
Treatment of a comminuted patella fracture with 3D printed hook plate implant: **a** 3D representation of the fracture; **b** 3D printed hook plate implant on 3D printed model of the patella (opposite side mirrored); **c** intraoperative status with reduction and temporary K‑wire fixation; **d** 3D printed patient-specific hook plate implant with screws placed

Models made from contralateral mirror images or digitally reduced fracture fragments can aid implant selection and patient-specific implant design [7, 15]. While it is recommended that fracture models be produced in the hospital for rapid turnaround, outsourced production is an acceptable alternative for elective indications (Fig. 3; [16, 17]).

## Infrastructure

### Current practice.

All six institutions produced most (> 51%) of their 3D models in-house. In four centres, prosumer grade printers (costing < €100,000) are the mainstream. Industrial grade 3D printers (costing > €100,000) are in use by two centres.

The most popular technology is fused filament fabrication (FFF) or fused deposition modelling (FDM). Acrylonitrile butadiene styrene (ABS) and polylactic acid (PLA) are common materials for FDM printers. Other technologies employed include powder bed selective laser sintering (SLS), PolyJet (PJ) and stereolithography (SLA). All centres outsourced their metal 3D printing tasks to commercial production partners for custom-made implants.

In four of the six surveyed centres, the 3D printing facilities are operated in the orthopaedics and traumatology unit. In two centres, 3D printing is run as a multispeciality collaborative “point-of-care” 3D printing service. Staffing of the facilities varied greatly. Three centres hired dedicated nonmedical personnel for 3D printing. In two of the six centres, surgeons do not take part in the routine workflow or management of the facilities. In one centre, 3D printing is wholly carried out by on-duty orthopaedic surgeons.

### Consensus.

All above 3D printing techniques have satisfactory dimensional accuracies within 0.2 mm for typical sized bone models [12]. FDM using ABS or PLA materials are suitable for fracture care with low setup cost, space utilization and material wastage. Print layer thickness between 0.4–0.6 mm balances between print speed and detail level. Industrial grade machines have higher reliability than prosumer grade machines. International Standard Organization ISO-10993 equivalent biocompatibility certification may be required to fulfil local regulations for on-table use. This standard is available for selected ABS materials. Both ABS and PLA products are not suitable for steam autoclaving at 132 °C and the surface details and durability maybe inferior to other technologies. Feedstock humidity can increase the risk of FDM print failures; thus they should be operated in dedicated dehumidified environments.

Surgical jigs produced by SLS using Nylon-12 material are both durable, autoclavable and suited for larger volume production where multiple cases are produced in one machine cycle. In small volume SLS runs, there is significant material wastage. SLA printers uses ultraviolet (UV) light to solidify liquid resins and is suitable for low volume production with low setup costs. A variety of SLA resins are available depending on the application. General SLA parts have high surface quality but are usually brittle. Specific high-temperature resistant resins are now available for surgical guide manufacturing.

All 3D printing processes require specialized postprocessing stations

PolyJet (PJ) technology also uses UV curing but differs with SLA where multiple materials and colours can be used for production of vivid anatomical models. The maintenance of PJ machines is manpower intensive as the print-heads are vulnerable to blockage and materials are generally more expensive. PJ technology is suitable for collaborative use with cardiovascular and surgical oncological applications.

Metal 3D printing powder is potentially explosive and hazardous. Furthermore the intensive postprocessing workflow, high setup-cost, large space requirements and lower usage mean that metal 3D printing devices are best installed at dedicated locations servicing multiple hospitals.

**Fig. 4:**
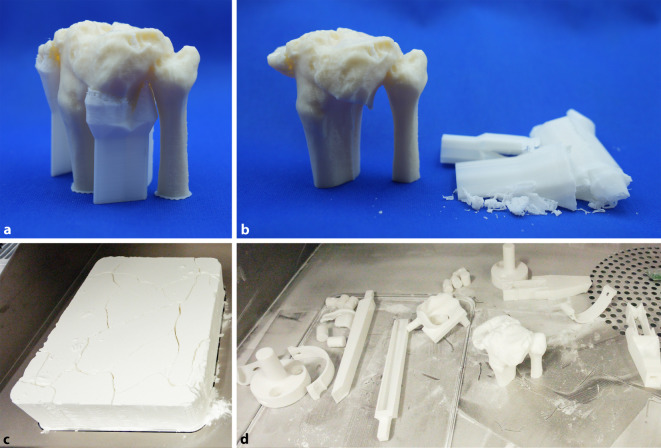
Tibial plateau fracture model in acrylonitrile butadiene styrene copolymer (ABS; Fortus450mc/ABS-M30i, Stratasys, Eden Prairie, MN, USA). **a**, **b** Removal of intramedullary support structures and in the cancellous fracture gaps can be very time consuming. **c**, **d** Powder fusion with selective laser sintering (SLS; P110/PA2200, EOS, Krailling, Germany) demonstrating production of multiple objects in a single machine cycle before (**c**)and after (**d**) powder removal

All 3D printing processes require significant manual postprocessing effort. Specialized postprocessing stations and dedicated space is needed. These tasks include support structure removal and cleaning. Close collaboration between physicians and technical staff appears to be the most efficient manpower arrangement. On-site dedicated staff proficient in image segmentation, anatomical knowledge and operation of 3D printers are necessary. Having orthopaedic surgeons trained in 3D printing skills can improve the utilization rate of the printing facility; however, unforeseen, emergency 3D printing orders may impede efficient running (Fig. 4).

## Image acquisition

### Current practice.

Fine-cut CT scanning of acute fractures are performed as a clinical routine in all centres. The decision for 3D printing is made after clinician review. Under this routine, no special arrangement with the radiology department is needed. For elective osteotomies, a dedicated fine-cut CT for digital planning and 3D printing is needed. Longer scans lengths to include the proximal and distal joints, and contralateral limbs are commonly needed. Direct retrieval of digital imaging and communications in medicine (DICOM) data from the picture archiving and communication system (PACS) network to the segmentation workstation is routine in only one surveyed institution. The remaining five institutions required manual export via compact disc or universal serial bus (USB) flash medium due to computer security rules. Data anonymization is routine in four of the six centres. Unique identification data is stamped on the 3D model to prevent misidentification in five centres.

Close collaboration with the radiology department regarding the scanning protocol is essential

Completion of an order form is required for two centres and written informed consent for 3D printing is required in one centre. Informal ordering and communication though smartphone chat apps are common for tracking of cases and specifying 3D printing requirements. Only one centre had a formalized job tracking process accessible to the ordering party. The typical wait time is 24 h for peri-articular limb fracture models, 24–48 h for pelvic and acetabulum fracture models and between 3 days to 2 weeks for osteotomy guides.

### Consensus.

Due to surgeons’ busy schedules, a lag in CT data retrieval and communication can significantly slow down the 3D printing workflow. A clinical protocol where 3D printing is ordered prior to the CT scan and images are transferred via computer network can improve the service efficiency. Consensus with the CT department regarding the scanning protocol is essential. Slice thickness, patient positioning, region of interest and metal artefact suppression tactics should be specified. Data deidentification is associated with extra work and risks mixing up of cases and should not be mandatory when 3D printing is performed in-house. Digital data and printed models should be tagged with patient initials or unique numbers to prevent misidentification. A digital data policy to protect patient privacy should be enforced when third parties are involved.

## Digital workflow

### Current practice.

All six centres have dedicated segmentation workstations installed. Commercial software is used by five hospitals and freeware is used by one hospital. Segmentation by Hounsfield unit thresholding is the preferred method in all six hospitals. Strategies to save material usage and print time includes routine cropping, filling of intramedullary space and size shrinkage to below 1:1 for pelvic and acetabulum models when implant contouring is not a concern.

The digital models and 3D models must be validated by surgeons

After office hours segmentation is offered in 3 centres. Mandatory checks for accuracy in the digital models are performed by surgeons before printing in four centres.

### Consensus.

**Fig. 5:**
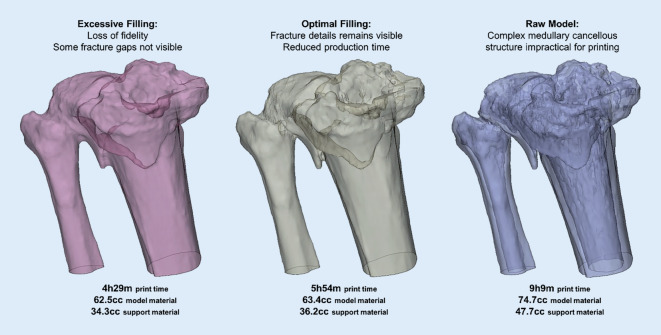
Fracture model fabrication requires a balance between preserving important fracture detail and removing complex intramedullary and cancellous structures. Figures show estimated production time and material usage using an industrial fused deposition modelling (FDM) printer (Fortus 450mc, Stratasys, Eden Prairie, MN, USA)

The digital workflow is technically demanding and requires trained staff [13]. Segmented digital models are suitable for printing only after optimization. The main optimization steps are trimming, hole filling and stamping of identity information. Overzealous filling of gaps and oversimplification of the model to facilitate the manufacturing process may lead to loss of fidelity and details. Training frontline physicians can facilitate off-hours printing for urgent cases. Patient-specific instruments require specialized personnel highly familiar with digital planning and the surgical procedure. The surgeon becomes more familiar with the treatment when personally involved in the digital workflow. Validation of digital models and plans should be carried out with surgeons before printing (Fig. 5).

## Production

### Current practice.

The operation of 3D printer, postprocessing and the selection of materials are handled by trained personnel in all centres surveyed. Printers are operated overnight unsupervised in all units. Technical support is available during non-office hours in two centres. None of the centres practiced routine quality and control checks by independent personnel after the production.

The typical fraction of time spent in procedures after availability of the CT scans varied between units. Importantly, delays in the ordering mechanism and data transfer before segmentation comprised between 15 and 50% of the overall production time.

A protocol-driven sterilization workflow with the theatre sterile surgical unit (TSSU) is enforced in 4 of 6 centres. A standardized model delivery and preoperative “timeout” checklist for identity, indication, laterality, and operative date is enforced in two centres. H_2_O_2_ plasma sterilization (70 °C) is the most popular method for FDM parts and steam autoclave (132 °C) is the most common sterilization for SLS-Nylon-12 and thermally stable parts. None of the units have a specific disposal and material recycling procedure. Commonly, the models are retained by the surgeons for staff training and display after clinical use.

### Consensus.

**Fig. 6:**
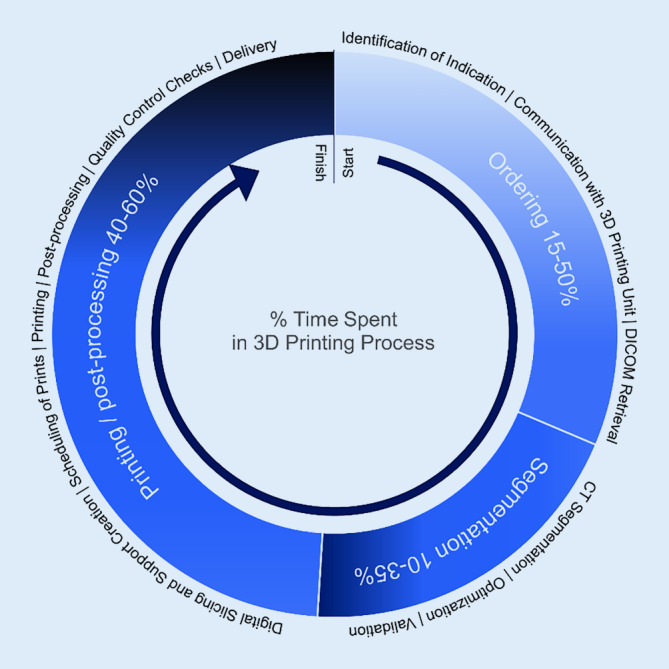
The three most time-consuming processes in clinical 3D printing

Logistical and communication delays significantly contributed to increased production time. Hiring dedicated staff with off-hours rotas, and clever scheduling of tasks can significantly improve speed and reliability. A standard operation protocol can streamline ordering, production, delivery and sterilization procedures. Fire safety mechanisms should not be overlooked when printers are operated overnight (Fig. 6).

## Organization and funding

### Current practice.

In three centres, the 3D printing facility is managed by the orthopaedics and trauma unit. Four centres had a hospital level multispeciality steering committee overseeing all aspects of clinical 3D printing. The production unit is physician led in four centres, and in two centres this is led by an engineer. One centre had weekly case review meetings and none of the centres had audit meetings. Four centres provided printing and delivery services to other hospitals on a regular basis.

In four centres, initial setup and running of 3D printing services is supported mainly by research funding. The hospital budget provides partial to full financial support in three centres. In one centre, a commercial entity finances the point-of-care service within the hospital premises. In five centres, there are no charging and insurance reimbursement mechanisms and patients therefore did not need to pay for 3D printing services.

### Conclusion.

The most established clinical uses of 3D printing are musculoskeletal, craniomaxillofacial [14, 15], neurosurgical [16] and cardiovascular interventions [17].

The radiology department plays a central role in providing high-quality imaging services. The multidisciplinary use of 3D printing in a hospital is termed “point-of-care” 3D printing. Routine implementation of 3D printing is cost efficient where there is a sufficient case load [18].

Multispecialty “point-of-care” 3D printing implemented at the hospital-wide level may improve resource utilization. There is no consensus on whether the 3D printing should be run as a point-of-care model or under the orthopaedic trauma unit. The manpower needed for one hospital’s 3D printing service seems to be limited to a handful of dedicated personnel. A “hub and spoke” model where high load tertiary centres provide services to smaller hospitals can enhance efficient resource usage and enhance development of technical skills [19].

Use of a “hub and spoke” model can improve resource utilization

Currently, a lack of standardized guidelines and regulations despite the obvious clinical advantages may deter support from hospital administrators. Setting up of a sound reimbursed financial model is one major challenge for the sustainability of 3D printing services.

## Future development and research

### Current practice.

In all centres, staff are trained in daily practice under supervision, seminars, exchanges and workshops and there are no requirements for staff accreditation in certified courses. The centres’ have short-term targets in improving financial structuring, staffing, administrative oversight, protocol standardization, radiology department partnership and image segmentation automation. Five centres reported active participation in protocol-driven prospective clinical studies on peri-articular fractures (patient-specific guides, pelvic acetabulum fractures, femoral neck fractures) and custom implants (e.g., for patella and clavicles).

### Consensus.

The use of 3D printing for trauma is rapidly evolving and recent studies with good levels of evidence have been reported [1, 20]. Proficient digital skills are demanded. Certification courses in clinical 3D printing can standardize training. Most clinical indications requiring 3D printing are rare complex pathologies and therefore it is challenging to gather sufficient sample sizes for high impact randomized studies. Standardized guidelines on the best practises are needed. Artificial intelligence may considerably simplify the convoluted digital segmentation and modelling process.

## Practical conclusion

- This article is a summary of how clinical 3D printing is conducted in six surveyed centres. Multiple processes can facilitate rapid and efficient 3D printing. The authors hope that the detailed presentation of current practices and consensus will enhance the acceptance and implementation of this technology for the betterment of patient care.
- The small number of hospitals included is the main limitation of this analysis. The best practices recommendations that are listed are anecdotal and have not been scientifically tested.
- Their implementation ought to vary depending on available assets and adherence to local regulations.

## Supplementary Information

Supplementary material: A table listing all surveyed items from the six centres

## Declarations

For this article no studies with human participants or animals were performed by any of the authors. All studies mentioned were in accordance with the ethical standards indicated in each case.

The supplement containing this article is not sponsored by industry.
