# Supplementary material for: 3D printing in fracture treatment: Current practice and best practice consensus
Source: Unfallchirurgie (Heidelb). 2022 Jul 11;125(Suppl 1):1–7. doi: 10.1007/s00113-022-01159-y (PMC9722822; doi:10.1007/s00113-022-01159-y)
Supplement: Supplementary file 1 — Supplementary material: A table listing all surveyed items from the six centres [file 113_2022_1159_MOESM1_ESM.pdf]

Supplementary Material: Data collected from surveyed centres

Institution	QHK	UHK	AMU	NUS	FAW	BAB
Total hospital 3D Printed Cases per Year	>200	100-200	>200	100-200	100-200	50-100
Cases of fractures per year	100-200	50-100	25-50	50-100	50-100	25-50
**Top indications**	Plateau<br>Pilon<br>Acetabulum<br>Reverse shoulder guides<br>Malunions	Plateau<br>Pilon<br>Distal Femur<br>Humerus periarticular<br>Reverse shoulder guides	Malunions<br>Patella<br>Periarticular fracture	Plateau<br>Acetabulum<br>Malunions<br>Oncology	Acetabulum<br>Pilon<br>Plateau<br>Malunions<br>High tibial osteotomy	Acetabulum<br>Clavicle<br>Malunions
**Infrastructure**						
Main 3D printing location	On site	On site	On site	On site	On site	On site
Most used 3D printer grade	Industrial (costing >€100,000)	Prosumer (costing <€100,000)	Prosumer	Industrial	Prosumer	Prosumer
No. of industrial grade printers	3	0	1	1	0	0
No. of prosumer grade printers	1	2	10	5	5	1
Most used technology (Material)	FDM (ABS)	FDM (PLA)	FDM (ABS)	PJ (Various)	FDM (PLA)	FDM (PLA)
Other technologies	SLS, PJ	SLA (Surgical guide resin)	Nil	FDM SLA	Nil	Nil
Metal printer	Outsourced	Outsourced	Outsourced	Outsourced	Outsourced	Outsourced
Operational unit	Orthopaedics Department	Prosthetist and Orthotist unit	Orthopaedics Department	Point-of-care Lab	Orthopaedics Department	Orthopaedics Department
No. of Dedicated non-medical staff	1	2	1	3	0	0
No. of 3D Trained medical staff	3	0	2	0	10	2
No. of non-dedicated supporting staff	2	1	1	0	1	0
Pilon/plateau fractures typical wait time	<24hrs	<24hrs	<24hrs	<48hrs	<24hours	<24hours
Acetabulum fractures typical wait time	<48hrs	<48hrs	<48hrs	<48hrs	<48hrs	<48hrs
PSI Jigs typical wait time	1 week	3 days	1 week	1 week	3 days	2 weeks (outsourced)
**Imaging**						
CT Scanning for fractures	Per routine	Per routine	Per routine	Per routine	Per routine	Per routine
CT Scanning for PSI jigs	By special order	By special order	By special order	By special order	By special order	By special order
Wait time for CT for fractures	<24hrs	<48hrs	<24hrs	<24hrs	<24hrs	<24hrs
Wait time for CT for jigs	3 months	3 months	1-2 days		<24hrs	<24hrs
3D Printing Ordering form filling required	No	Yes	No	Yes	No	No
Patient consent	Not required	Not required	Not required	Written	Verbal	Not required
Main mode of ordering	Informal communication	Electronic ordering	Informal communication	Written forms	Informal communication	Informal communication
Job tracking process	No	No	No	Yes	No	No
DICOM transfer process	Media	Media	Media	Media	Local Network	Media
Anonymization	No	No	Routine	Routine	Routine	Routine
Patient Initials on 3D models	Yes	Yes	No	No	No	No
Identification number on 3D models	No	Yes	Yes	Yes	Yes	No
**Digital workflow**						
No. of segmentation workstations	2	1	2	1	3	1
Software	Commercial	Commercial	Commercial	Commercial	Commercial	Freeware
Segmentation technique	Thresholding	Thresholding	Thresholding / Semi-Automatic	Thresholding	Thresholding	Thresholding
STL optimizations before printing	Mandatory	Mandatory	Mandatory	Mandatory	Mandatory	Mandatory
Routine removal of IM canals	Yes	Yes	No	Yes	No	No
Routine cropping	Yes	Yes	Yes	Yes	Yes	Yes
Routine 1:1 size printing	Yes	Yes	Yes	Yes	Yes	Yes
Surgeons can perform segmentation and modelling	Yes	No	Yes	Yes	Yes	Yes
Off hours segmentation and modelling services	Yes	No	No	No	Yes	Yes
Digital validation by clinician	Yes	No	Yes	No	Yes	Yes
**Production**						
3D Printer operator	Technical Staff	Prosthetist	Postgraduate Research Student	Engineer	Surgeon	Surgeon
Off hours printing	Yes	Yes	Yes	Yes	Yes	Yes
Off hours technical support	Yes	No	No	No	Yes	No
Material selection	By printer operator	By printer operator	By printer operator	By printer operator	By printer operator	By printer operator
Independent post-production quality check	No	No	Yes for PSI jigs	No	No	No
Sterilization protocol	Yes	Yes	Yes	No	Yes	No
Preferred sterilization for low temperature (ABS/PLA) parts	H_2O_2 Plasma	H_2O_2 Plasma	Not done	H_2O_2 Plasma	Antiseptic soaking	H_2O_2 Plasma
Preferred sterilization for high temperature parts	Autoclave	Autoclave	Autoclave	Autoclave	N/A	Sterilized by manufacturer
ISO 10993 equivalent biocompatibility certificate required	Yes	Yes	No	Yes	No	No
%Time spent from scan to segmentation	50%	30%	20%	50%	15%	50%
%Time spent for segmentation	10%	10%	20%	10%	35%	10%
%Time spent for production and delivery	40%	60%	60%	40%	50%	40%
**Administration and funding**						
Service targets	Multispeciality point-of-care	Multispeciality point-of-care	Orthopaedics	Multispeciality point-of-care	Orthopaedics	Orthopaedics
Leadership	Clinician	Engineer	Clinician	Engineer / Clinician	Clinician	Clinician
Radiology Department Oversight	No	No	No	No	No	No
Multidisciplinary Steering Committee	Yes	Yes	Yes	Yes	No	No
Case review meetings	Yes	No	No	No	No	No
Audit meetings	No	No	No	Yes	No	No
Delivery to other hospitals	Yes	Yes	No	Yes	No	No
Main Funding Source	Research Funding	Hospital budget	Research Funding	Commercial Partnership	Research Funding	Research Funding
Patient payment / Insurance Reimbursement	No	No	No	Yes	No	Yes (Custom Implants only)
Written Standard Operating Procedures	No	Yes	Yes	Yes	No	No
Reimbursement from hospital budget	No (Applying)	Yes	No	Yes	No (Applying)	No
**Future development and research**						
Future improvement targets	Automated segmentation<br>Charging mechanism	Setup of admin structure<br>Staffing	Charging mechanism	Physician awareness	Charging mechanism	Charging mechanism<br>Funding
Protocol driven prospective research projects	Articular fractures RCT<br>Custom implants	Pilon fracture	Patella custom implants	None	Acetabular PSI guides<br>Femoral neck PSI guides<br>Custom implants	Custom clavicle plates
Other active research	Case series	Case series	Case series	Case series	Case series<br>Massive defect reconstruction	Case series<br>Acetabulum training study
